# Monitoring Mitochondrial Function in *Aedes albopictus* C6/36 Cell Line during Dengue Virus Infection

**DOI:** 10.3390/insects12100934

**Published:** 2021-10-14

**Authors:** María E. Santana-Román, Paola Maycotte, Salvador Uribe-Carvajal, Cristina Uribe-Alvarez, Nayeli Alvarado-Medina, Mohsin Khan, Aleem Siddiqui, Victoria Pando-Robles

**Affiliations:** 1 Centro de Investigaciones Sobre Enfermedades Infecciosas, Instituto Nacional de Salud Pública, Cuernavaca 62100, Mexico; santanaromanelizabeth@gmail.com (M.E.S.-R.); nalvmed@gmail.com (N.A.-M.); 2 Centro de Investigación Biomédica de Oriente, Instituto Mexicano del Seguro Social, Puebla 74360, Mexico; bisbenzimida@gmail.com; 3 Instituto de Fisiología Celular, Universidad Nacional Autónoma de México, Mexico City 04510, Mexico; suribe@ifc.unam.mx (S.U.-C.); cristina.uribe-alvarez@fccc.edu (C.U.-A.); 4 Division of Infectious Diseases, Department of Medicine, University of California, San Diego, CA 92093, USA; mohsinshaz1@gmail.com (M.K.); asiddiqui@health.ucsd.edu (A.S.)

**Keywords:** dengue virus, *Aedes aegypti*, mitochondria

## Abstract

**Simple Summary:**

Dengue is an important and growing public health problem. To date, no specific therapeutic or effective prophylactic measures exist. Therefore, vector control remains the primary approach to prevent dengue virus (DENV) infection in humans. Recent findings highlight that viruses regulate mitochondrial function and dynamics to facilitate viral proliferation. In this study, we report that DENV infection modulates mitochondrial physiology in C6/36 mosquito cells. Our results revealed that DENV alters redox metabolism and mitochondrial membrane potential without any significant change in cellular ATP pool or viability. In addition, we observed preservation of the respiratory control ratio and translocation of mitofusins to mitochondria. These results suggest that mitochondrial fusion could be required for the maintenance of mitochondrial function in C6/36 mosquito cells infected with DENV.

**Abstract:**

*Aedes aegypti* and *Aedes albopictus* mosquitoes are responsible for dengue virus (DENV) transmission in tropical and subtropical areas worldwide, where an estimated 3 billion people live at risk of DENV exposure. DENV-infected individuals show symptoms ranging from sub-clinical or mild to hemorrhagic fever. Infected mosquitoes do not show detectable signs of disease, even though the virus maintains a lifelong persistent infection. The interactions between viruses and host mitochondria are crucial for virus replication and pathogenicity. DENV infection in vertebrate cells modulates mitochondrial function and dynamics to facilitate viral proliferation. Here, we describe that DENV also regulates mitochondrial function and morphology in infected C6/36 mosquito cells (derived from *Aedes albopictus*). Our results showed that DENV infection increased ROS (reactive oxygen species) production, modulated mitochondrial transmembrane potential and induced changes in mitochondrial respiration. Furthermore, we offer the first evidence that DENV causes translocation of mitofusins to mitochondria in the C6/36 mosquito cell line. Another protein Drp-1 (Dynamin-related protein 1) did not localize to mitochondria in DENV-infected cells. This observation therefore ruled out the possibility that the abovementioned alterations in mitochondrial function are associated with mitochondrial fission. In summary, this report provides some key insights into the virus–mitochondria crosstalk in DENV infected mosquito cells.

## 1. Introduction

Mitochondria are highly dynamic and inter-connected organelles that regulate a variety of cellular processes including energy homeostasis, redox status, thermogenesis, and cell death via apoptosis [1]. As the “powerhouse of the cell”, mitochondria are responsible for up to 90% of cellular adenosine triphosphate (ATP) production by oxidative phosphorylation (OXPHOS), driven by ATP synthase and the electron transport chain (ETC) found in the inner mitochondrial membrane; where electrons are passed along complexes I/II, III, and IV [2]. The energy released by this electron transfer is used to pump protons across the inner mitochondrial membrane, generate an electrical potential and a proton gradient resulting in the establishment of the mitochondrial membrane potential (MMP). The MMP is a component of the overall proton motive force that drives ATP production in mitochondria [3]. During the energy conversion process, reactive oxygen species (ROS) are produced. The mitochondrial ETC carries out a series of redox reactions, where electrons may escape mainly from complexes I and III and react with molecular oxygen to form superoxide anion (O_2_^•−^), which can later be transformed into hydrogen peroxide (H_2_O_2_), and hydroxyl radicals (•OH), or oxygen singlet (^1^O_2_) [4,5].

On the other hand, the mitochondrial cycle involves fusion, fission, and mitophagy, processes that control the number and size of mitochondria. Fusion (combination of two organelles into one) is mediated by three large GTPases of the Dynamin superfamily, Mitofusins 1 and 2 (MFN1 and MFN2) and Optic atrophy 1 (OPA1) [6]. Fission (the division of a single organelle into two) is facilitated by Dynamin-related protein 1 (Drp1), while mitophagy (degradation of damaged organelles) is mediated by Parkin and Pink proteins [7]. The main reason for continual mitochondrial dynamics is to prevent the accumulation of dysfunctional organelles to maintain cell homeostasis.

Dengue is the most prevalent *Aedes*-borne viral disease that places a heavy socioeconomic and disease burden on many tropical and subtropical countries. DENV is transmitted to humans principally by *Ae. aegypti* and *Ae. albopictus* mosquitoes [8]. DENV is an enveloped virus belonging to the Flavivirus genus of the *Flaviviridae* family. The genome is a positive-sense single-stranded RNA of 10.7 kb translated as a single polyprotein and subsequently cleaved into three structural proteins (capsid-C, envelope-E, and pre-membrane-prM) and seven nonstructural (NS) proteins (NS1, NS2A/B, NS3, NS4A/B, and NS5) [9]. DENV replication takes place on modified endoplasmic reticulum (ER) membranes [10]. Following RNA replication, synthesis of the viral proteins and immature virions are assembled inside the lumen of the ER. During this process, the virus particles obtain their enveloped, mature lipid bilayer by passing through the Golgi and trans-Golgi network. Finally, progeny virus particles are released from the cell via exocytosis [11].

DENV infection modulates mitochondrial dynamics in mammalian cells to regulate the innate immune signaling and promote virus replication. In hepatic cells (Huh-7), DENV protein NS4B induced mitochondrial elongation due to a reduction in Drp1 protein levels in mitochondria [12,13]; also, DENV produces an increase in cellular respiration and decreases ATP production [14]. Meanwhile, in A549 lung cells, the viral protease NS2B3 cleaved mitofusins (MFN1 and MFN2) and impaired mitochondrial dynamics [15].

To expand our knowledge about the role of mitochondria during DENV infection in the mosquito vector, we evaluated mitochondrial function in DENV infected C6/36 mosquito cell line. Here, we show that upon DENV infection, an increase in ROS levels and MMP occurs in infected mosquito cells. Our results highlight a fine modulation of mitochondrial function during DENV infection in the C6/36 cell line. In addition, we offer the first evidence that DENV induces the translocation of mitofusins (MFNs) to mitochondria.

## 2. Materials and Methods

### 2.1. Cells and Virus

C6/36 cell line (derived from mosquito *Ae. albopictus*) and Vero-E6 (African green monkey *Cercopithecus aethiops* kidney epithelial cells) were kindly donated by Dr. Rosa María Del Angel from CINVESTAV, Mexico. C6/36 cells were maintained in L-15 medi-um (Invitrogen, Carlsbad, CA, USA) with 10% fetal bovine serum (FBS), 10% triptose (SIGMA-Aldrich) and 1% antibiotic-antimycotic (Invitrogen, Carlsbad, CA, USA) at 28 °C in a closed system. Vero E6 cells were maintained in DMEM containing 10% FBS at 37 °C under 5% CO_2_ atmosphere.

Dengue virus serotype 2 (DENV2, New Guinea C strain) was donated by the Instituto Nacional de Diagnóstico y Referencia Epidemiológica-Mexico (INDRE) and propagated in the brain of neonatal BALB/c mice (2–3 days old). Mice were monitored daily until signs of infection were observed, such as ataxia, clumsiness, slow movement, and partial or total paralysis. Afterward, mice were sacrificed and their brains were extracted. The brain extracts were sonicated for 6 cycles at 40 Hz, then centrifuged at 10,000× *g* for 30 min at 4 °C, the supernatant was removed, filtered, and stored at −70 °C.

DENV2 was propagated in C6/36 cells, and the virus titers were determined by focus-forming assay in Vero E6 cells as previously described [16].

For infection assay and monitoring, 80% confluent monolayers of C6/36 cells in 25-cm^2^ tissue culture flask were infected with DENV2 at MOI 5 for 1 h at 28 °C, then they were washed three times with PBS and the infection was allowed to proceed for different periods of time according to the experiment.

### 2.2. ROS Measurement

ROS levels were measured in mock and DENV2-infected C6/36 cells using 2′, 7′-dichlorofluorescein diacetate (H_2_DCFDA, Cellular Reactive Oxygen Species Detection Assay Kit, Abcam, Cambridge, UK) following the manufacturer’s instructions. The experiments were performed on 96-well plates 40,000 cells were seeded per well and infected with DENV2 at MOI 5. Briefly, cells were labeled with 5μM H_2_DCFDA for 45 min. Afterwards, the cells were washed once with PBS. H_2_DCFDA is deacetylated by cellular esterase to a non-fluorescent compound, which is later oxidized by ROS into 2′, 7′-dichlorofluorescein (DCF). The fluorescence of DCP was detected at Ex/Em = 485/535 nm in a FLUOstart Omega instrument (BMG Labtech, Ortenberg, Germany). The ROS generator t-Butyl hydroperoxide (TBHP) was used as a positive control.

### 2.3. Cell Viability Assay

Cell viability was determined with the Cell Growth Determination kit (Sigma-Aldrich, St. Louis, MO, USA) following the manufacturer’s instructions. The experiments were carried out on 96-well plates (Corning, NY, USA), 40,000 cells were seeded per well and infected with DENV2 at MOI of 5. After different hours post-infection, we used 50 μg of 3-(4, 5-dimethylthiazol-2-yl)-2,5-diphenyltetrazolium bromide (MTT) per sample. The MTT assay involves the conversion of the water-soluble yellow dye MTT to an insoluble purple formazan by the action of mitochondrial reductase. Formazan is then solubilized in isopropanol and the concentration determined by optical density at 570 nm in an ELISA-type plate reader (IMarkTM, Bio-Rad, Hercules, CA, USA). 

### 2.4. ATP Measurement

The experiments were performed with 40,000 cells per well on 96-well plates (Corning, Corning, NY, USA) mock and DENV2 infected at MOI of 5. ATP levels were measured by the Luminescent ATP Detection Assay kit (ab113849, Abcam, Cambridge, UK) according to the manufacturer’s instructions. The protocol involves lysis of the cell sample, addition of luciferase enzyme and luciferin to form oxyluciferin, compound luminescent that was measured in a FLUOstart Omega instrument (BMG Labtech, Ortenberg, Germany). 

### 2.5. Mitochondrial Membrane Potential Assay

The experiments were performed with 40,000 cells per well on 96-well plates (Corn-ing, Corning, NY, USA) mock and DENV2 infected at MOI of 5. To measure mitochondrial membrane potential at different hours post-infection, the TMRE-MMP Assay Kit (ab113852, Abcam, Cambridge, UK) was used according to the manufacturer’s instructions. Tetramethylrhodamine ethyl ester (TMRE) is a cell permeant, positively-charged, red-orange dye that readily accumulates in active mitochondria due to their relative negative charge. The assay was done with 200 nM of TMRE per well and the fluorescence was measured at Ex/Em = 549/575 nm in a FLUOstart Omega instrument (BMG Labtech, Ortenberg, Germany). As control positive, we used the FCCP (Carbonyl cyanide 4-(trifluoromethoxy) phenylhydrazone), a proton ionophore that acts as an uncoupler of oxidative phosphorylation (OXPHOS).

### 2.6. Oxygen Consumption Measurement

Oxygen consumption was measured in a high-resolution Clark-oxygraph (Oroboros instrument, Innsbruck, Austria) in a 1.0 mL water jacket maintained under constant stirring at 28 °C. The chemicals were purchase from Sigma-Aldrich (St. Louis, MO, USA). 

**Figure 1:**
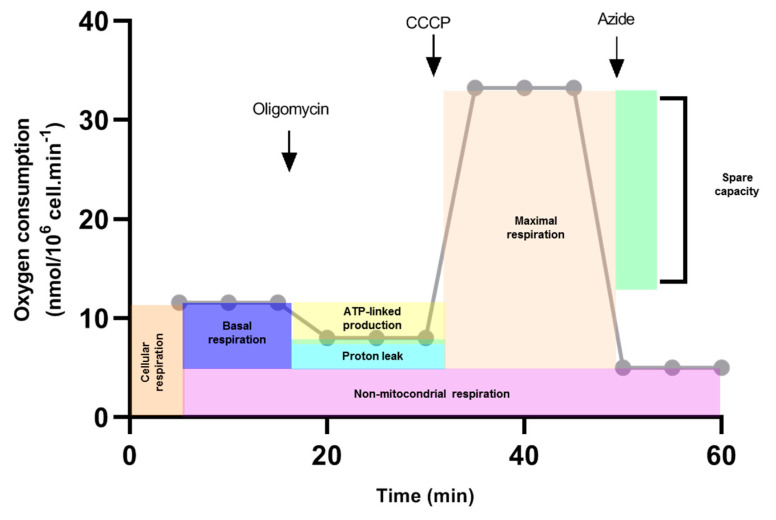
Calculations of mitochondrial function (Theorical example). Oxygen consumption rate was measured in response to sequential injections of oligomycin, CCCP and sodium azide (arrows). First, cellular respiration was evaluated. Oligomycin was added and oxygen consumption was traced. This record is used to calculate ATP-linked production and proton leak. The CCCP proton ionophore collapses the inner membrane gradient, which drives the electron transport chain to function at its maximum rate (Maximal respiration). Non-mitochondrial respiration was calculated when sodium azide was added. Basal respiration was estimated by subtracting the non-mitochondrial respiration of cellular respiration. Spare capacity was determined by subtracting basal respiration from maximal respiration.

Permeabilized C6/36 cells were used for analyses of respiratory properties. Then, 1 × 10^6^ cells were re-suspended in respiration buffer (100 mM KCl, 200 mM Tris-HCl, 10 mM KH_2_PO_4_, 75 mM mannitol) and 2.5 mM of ADP, 0.5 mM succinate as a complex II substrate and 2.2 mM of cytochrome c were added before the measurements. The measurements were made with 25 μg of digitonin as a permeabilizing agent, and three records were made with: 5 μg oligomycin as an inhibitor of ATP synthase, 200 nM CCCP as an uncoupling agent (carbonylcyanide-3-chlorophenylhydrazone) and 2 mM sodium azide, which is a complex IV inhibitor (Table 1). After recording cellular respiration, oligomycin was added to record oxygen consumption that represents the proton leak through the inner mitochondrial membrane. Maximum respiration was achieved by adding CCCP 200 nM. The block of respiration observed after the addition of sodium azide reveals the non-mitochondrial oxygen consumption in C6/36 cells. From this oxygen record, we calculated cellular respiration, non-mitochondrial respiration, basal respiration, proton leak, maximal respiration, spare respiratory capacity, and coupling efficiency (Figure 1) to evaluate mitochondrial function [17].

### 2.7. Immunofluorescence

The C6/36 cells were grown on glass coverslips, fixed in 4% paraformaldehyde, permeabilized with 0.25% TritonX-100, and incubated with primary antibodies: rabbit polyclonal antisera recognizing the drosophila MFNs and Drp1 have been previ-ously described [18,19] (one aliquot of each of these antibodies was kindly donated by Alexander Whitworth, University of Sheffield and Leo Pallanck, University of Washington, respectively), mouse anti-DENV (Santa Cruz), and rabbit anti-uncoupling protein 2/UCP2 (Cell Signaling). As secondary antibodies were used, anti-mouse IgG-Alexa Fluor 488 (Thermo Scientific, Waltham, MA, USA), donkey anti-rabbit IgG-Alexa Fluor 594 (Thermo Scientific), and donkey anti-goat IgG-Alexa Fluor 647 (Thermo Scientific) were used. DAPI was used for nuclei staining. Images were visualized under a 100× oil objective using a FluoView 1000 confocal microscope (Olympus, Tokyo, Japan).

### 2.8. Statistical Analysis

Data in graphs are presented as bars with standard deviations (SD). Comparison between groups was done by Kruskal–Wallis test for non-parametric data followed by Dunn’s multiple comparison test and by ANOVA followed by Sidak’s or Dunnett’s multiple comparison tests for parametric data. Differences of *p* < 0.05 were considered significant. The graphs and analysis were done in GraphPad Prism version 4.00.

## 3. Results

### 3.1. DENV2 Infection Induces Oxidative Stress in C6/36 Cells 

Under physiological conditions, ROS are produced at low levels and act as secondary messengers to regulate diverse biological processes. However, an imbalance between oxidation and antioxidant systems can cause tissue damage, inflammation and cell death [20]. In insects, ROS have been shown to modulate fecundity [21], immune response [22] and vector competence in the *Anopheles–Plasmodium* interaction [23].

**Figure 2:**
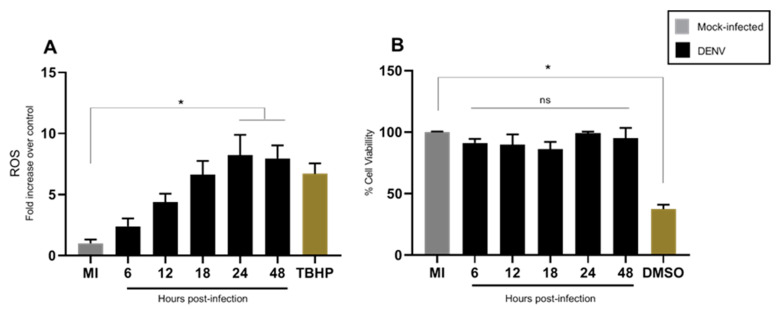
DENV2-infection induced high ROS levels in C6/36 cell line without affecting cell viability. C6/36 cells were infected with DENV2 at MOI 5 and incubated for different periods of time—6, 12, 18, 24, and 48 h post-infection. (**A**) ROS were measured with 5μM H2DCFDA. Data are expressed as fold increases over mock infected cells at each time point; TBHP was used as a positive control. (**B**) The cell viability was measured with MTT assay, the results are expressed as percentage of that of mock infected control; as a positive control 10% *v*/*v* of DMSO was used 12 h before treatment with MTT. All data are expressed as the mean ± SD obtained from at least three independent experiments. Significance was determined by Kruskal–Wallis test followed by Dunn´s multiple comparison test. *p* < 0.05 (*). ns: non-significant; MI: mock-infected; TBHP: tert-Butyl hydroperoxide; DMSO: dimethylsulfoxide; DENV: dengue virus.

To determine the intracellular ROS levels during DENV2 infection in C6/36 cells, we used a fluorescent dye H_2_DCFDA. Our result showed increased ROS levels between 6 to 48 hpi in comparison to non-infected cells at the same time (Figure 2A). The maximum eight-fold higher peak was detected at 24 hpi (*p* < 0. 05). High ROS levels can affect cell survival and therefore we next measured cell viability. We observed no significant difference in viability at 24 or 48 hpi (*p* < 0.05) (Figure 2B). These data suggest that mosquito cells infected with DENV showed an imbalance in their cellular redox state without any difference in cell survival throughout the period of observation (0–48 hpi). This finding is in agreement with previous reports [24]; although in our case, the ROS level showed a higher increase (8-fold at 24 hpi) and this difference could be due to infection conditions. All these observations seem critical for DENV in C6/36 cells and have not previously been defined.

### 3.2. DENV2 Infection Does Not Alter the General Energetic Status of C6/36 Cells

The maintenance of ATP levels is a requisite for normal cell function and survival. Otherwise, cells with a low MMP are committed to undergo apoptosis, whereas those with a high MMP are capable of exiting the apoptotic pathway [25]. As an approach to explore if mitochondria were responsible for maintaining cell viability, we evaluated MMP by TMRE and the bioenergetic status was measured by total cellular ATP levels using a chemiluminescence method.

**Figure 3:**
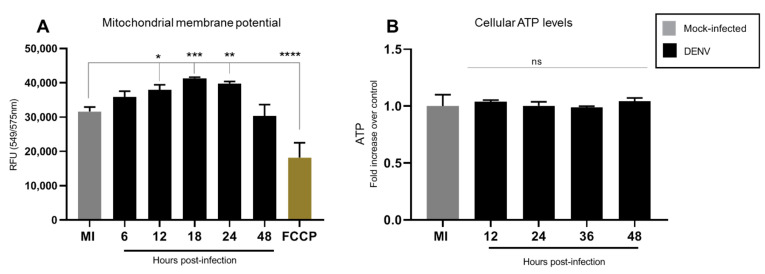
DENV2 infection induced a slight increase in MMP without affecting cellular ATP content. C6/36 cells were infected with DENV2 at MOI 5 and incubated for different periods of time 6, 12, 18, 24, and 48 hpi. (**A**) MMP was measured with TMRE-mitochondrial membrane potential assay at different times post-infection. Results were normalized to mock infected cells at the same time point after infection; FCCP was used as a positive control. Data represent mean ± SD of three independent experiments. Significance was determined by one-way ANOVA test followed by Dunnett´s multiple comparison test. *p* < 0.05 (*), *p* < 0.001(**), *p* < 0.0001 (***) and *p* < 0.00001 (****). (**B**) ATP levels were measured with the bioluminescence assay at different times post-infection. Data represent mean ± SD of three independent experiments. Significance was determined by a Kruskal–Wallis test followed by Dunn´s multiple comparison test. MMP: mitochondrial membrane potential; DENV: dengue virus; FCCP: Carbonyl cyanide-p-trifluoromethoxyphenylhydrazone; ns: non-significant.

Our results showed a slight increase in MMP between 12 and 24 hpi, which returned to basal levels at 48 hpi (Figure 3A). Total cellular ATP levels in DENV infected C6/36 cells remained unchanged as compared to uninfected control (Figure 3B). This indicates that DENV does not alter total cellular energy homeostasis in C6/36 mosquito cells.

### 3.3. Mitochondrial Respiration in C6/36 Cells during DENV2 Infection

**Figure 4:**
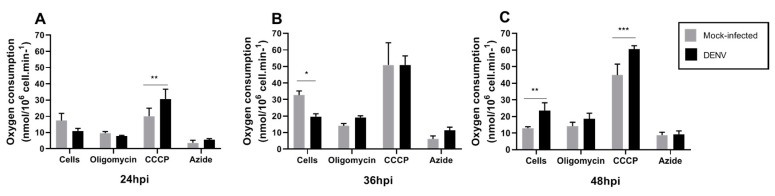
Typical profile of oxygen consumption in C6/36 mock and DENV2-infected cells. C6/36 cells were infected with DENV2 at MOI 5 and incubated at different time points of 24 hpi (**A**), 36 hpi (**B**), and 48 hpi (**C**). Oxygen consumption trace was determined using a high-resolution Clark-oxygraph in a 1.0 mL water jacket maintained under constant stirring at 28 °C, sequential injections of oligomycin, CCCP, and sodium azide were added. These chemicals act as an ATP synthase inhibitor, mitochondrial uncoupler, and inhibitor of complex IV, respectively. Data are shown as mean ± SD from three independent experiments. Statistical analyses were performed by two-way ANOVA and Sidak´s multiple comparison test. *p* < 0.05 (*), *p* < 0.001(**), *p* < 0.0001 (***). MI: mock-infected; CCCP: carbonyl cyanide m-chlorophenyl hydrazone; hpi: hours post-infection; DENV: dengue virus.

The results described above indicate alterations in mitochondrial function and ROS production without consequences in total cellular ATP levels. Since changes in MMP have been associated with oxygen consumption and ROS production, we assayed the mitochondrial respiration profile targeting different complexes of ETC by pharmacological inhibitors. We evaluated mitochondrial respiration on DENV infected C6/36 mosquito cells for 48 h. Oxygen consumption rate (OCR) was measured with Clark-electrode in permeabilized cells and we used mitochondrial inhibitors and substrates (Table 1) to evaluate mitochondria functions. All measures were done with succinate as a substrate for complex II and we added ADP and cytochrome c. Changes in oxygen consumption were observed with CCCP treatment at 24 and 48 hpi, while cellular oxygen consumption (cells) was altered at 36 and 48 hpi (Figure 4). These findings suggest important changes in cellular and mitochondrial oxygen consumption caused by DENV infection.

**Figure 5:**
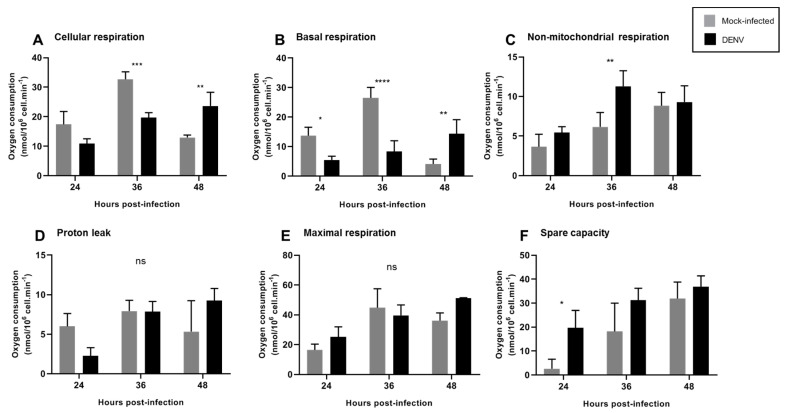
DENV2 induced changes in mitochondrial function of C6/36 cell line. C6/36 cells were mock and DENV2-infected at MOI 5 at different times post-infection, and respiration parameters were determined. (**A**) Cellular respiration was measured before the injection of any inhibitors. (**B**) Basal mitochondrial respiration. (**C**) Non-mitochondrial respiration was obtained after azide injection. (**D**) Proton leak. (**E**) Maximal respiration. (**F**) Spare capacity. Data are shown as mean ± SD from three independent experiments. Statistical analyses were performed by two-way ANOVA and Sidak´s multiple comparison test. *p* < 0.05 (*), *p* < 0.001 (**), *p* < 0.0001 (***), *p* < 0.00001 (****). ns: non-significant; DENV: dengue virus.

In order to specifically assess changes in mitochondrial function, cellular respiration, non-mitochondrial respiration, basal respiration, proton leak, maximal respiration, spare respiratory capacity and coupling efficiency were calculated [17] (Figure 5).

We observed a decrease in cellular and basal mitochondrial respiration at 36 hpi, which was reestablished after 48 hpi (Figure 5A,B). These findings are consistent with an increased MMP (Figure 3A) starting at 12 hpi, probably suggesting that decreased oxygen consumption is due to increased MMP. At 36 hpi, we observed an increase in non-mitochondrial respiration (Figure 5C), probably reflecting oxygen consumption from non-mitochondrial sources, likely cellular oxidases that could contribute to ROS production at this time point. Importantly, a slight but non-significant decrease in proton leak was observed at 24 hpi, which returned to basal levels at 36 hpi. Increased proton leak has been related to mitochondrial uncoupling and dysfunction [17] and to changes in proton leakiness across the mitochondrial inner membrane or to changes in MMP whereas decreased proton leak is related to mitochondrial fitness. No changes in maximal respiration were observed at the times tested and increased spare mitochondrial capacity was observed at 24 hpi, probably indicating the cellular response to infection, since this measurement reflects the capability of the cell to respond to an energetic demand.

**Figure 6:**
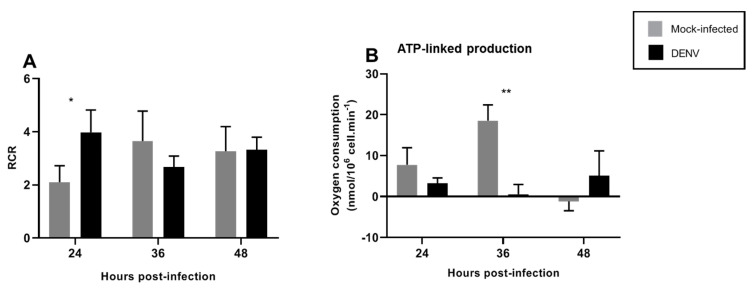
DENV2-induced changes in mitochondrial respiration in the C6/36 cell line. C6/36 cells were infected with DENV2 at MOI 5 and incubated for different periods of time; 24, 36, and 48 hpi. (**A**) The RCR (respiratory control ratio) represents the mitochondrial coupling state. (**B**) The ATP-linked production is the respiration contributing to ATP generation and was obtained by subtracting proton leak respiration from basal respiration. Data are shown as mean ± SD from three independent experiments. Statistical analyses were performed by two-way ANOVA and Sidak´s multiple comparison test. *p* < 0.05 (*), *p* < 0.001 (**). DENV: dengue virus.

The RCR is a useful measure to determine the integrity of the inner mitochondrial membrane and coupling of the electron transport chain in the synthesis of ATP. The RCR was calculated by divide CCCP (state 3u) between oligomycin (4o) (3u/4o). Increased RCR was observed upon DENV infection at 24 hpi, returning to basal levels after 36 hpi (Figure 6A). Additionally, ATP-linked production decreased at 36 hpi and returned to normal levels after 48 hpi (Figure 6B).

In mammalian cells, an increase in cellular respiration, decreased membrane potential, uncoupled mitochondria, and decreased energy charge in DENV-infected hepatic cells has been observed. Additionally, increased proton leak causing apoptosis has been described for DENV-infected HepG2 cells [12].

### 3.4. Changes in Mitochondrial Function Are Not Related to Mitochondrial Fission

Some viruses modulate mitochondrial dynamics to promote persistent infection and to attenuate the innate immune response [26]. Therefore, mitochondrial morphology can regulate the respiratory rate and enhance fusion, whereas fission decreases respiratory function [27]. To assess if mitochondrial dynamics changed during DENV2 infection in mosquito C6/36 cells, we evaluated the localization of Drp1 and MFNs, proteins involved in mitochondrial fission and fusion, respectively.

**Figure 7:**
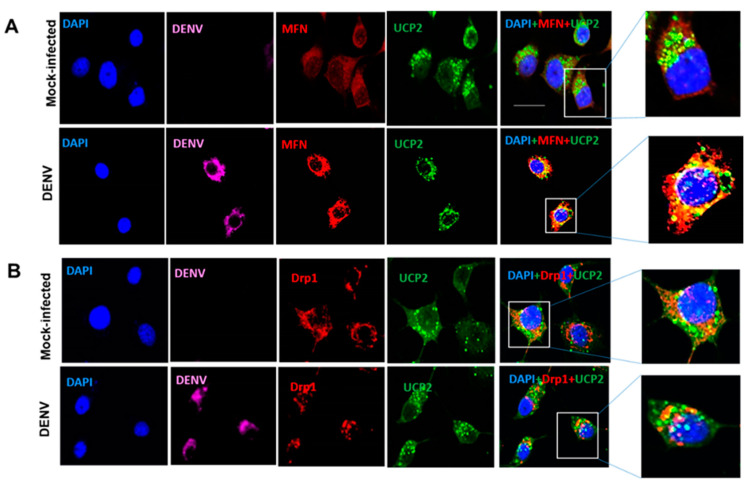
DENV2 induced MFNs translocation into the mitochondria of C6/36 cells. All cells were infected and mock-infected with DENV2 at an MOI of 10 and fixed at 36 hpi. Mitochondria were visualized by anti-UCP2 immunostaining and nuclei were visualized by DAPI stain (**A**) Colocalization of MFN and UCP2 in DENV infected mosquito cells (C6/36). MFN shown in red, UCP2 in green, and DENV in magenta. Magnified images of boxed area show merged fluorescence of MFN and UCP2 (yellow). (**B**) Drp1 does not colocalize with UCP2. Drp1 shown in red, UCP2 in green, and DENV in magenta. The insets show magnifications of the selected zones. The images were taken with an Olympus FluoView 1000 confocal microscope (scale bar, 20 μm). Representative images of three independent experiments are presented. MFN: mitofusins. Drp1: dynamin-related protein 1. UCP2: uncoupling protein 2. DENV: dengue virus.

Immunofluorescence analysis showed that MFNs translocate to the mitochondria in DENV2 infected cells, and colocalize with UCP2, an uncoupling protein proton transporter family member (UCPs) present in the mitochondrial inner membrane. While in mock-infected cells, MFNs localized to the cytoplasm (Figure 7A). Relative to uninfected cells, Drp1 translocation to mitochondria remained unchanged (Figure 7B). Both results suggest that mitochondrial fusion could be responsible for the maintenance of mitochondrial functions in C6/36 cells infected with DENV2, while no changes in Drp1 localization suggestive of mitochondrial fission were observed.

## 4. Discussion

Despite the importance of hematophagous female mosquitoes *Aedes* spp. as a vector of different arboviruses including DENV, most of our knowledge on dengue biology comes from studies in mammalian cells and limited work has been conducted in mosquitoes. Thus, vector–virus crosstalk and mitochondrial physiology during arbovirus infection remain poorly explored in the invertebrate host.

Several studies have shown that the cellular responses of DENV infection are markedly different between mammalian and insect cells. While apoptosis is a common consequence of mammalian cell infection [12], infected insect cells such as C6/36 cells can be maintained in culture for long periods [28]. In vitro studies also suggest that DENV-infected mosquito cells can survive through the antioxidant defense and antiapoptotic effects [24]. However, the oxidative stress level is critical for the control of both antiviral and apoptotic programs in DENV-infected cells [29]. Furthermore, ROS are important effectors of the immune system in insects. In *Anopheles* mosquitoes, ROS production limits *Plasmodium* infection, modulating MMP [30,31]. In *Drosophila*, bacterial infection has been shown to induce ROS generation by dDuox and was responsible for the oxidative damage to kill ingested microbes [22,32]. Studies have also shown that fine-tuned ROS production by Duox and Mesh contributed to maintaining homeostasis in the gut microbiome in *Ae. aegypti*. Likewise, ROS contributed to managing healthy gut–microbe interactions in insects [33,34]. Additionally, Wolbachia-infected *Ae. aegypti* is resistant to DENV infection through induction of oxidative stress, activation of the Toll pathway, antimicrobial peptide expression, and an antioxidant response [35].

Our results also showed an eight-fold increase in ROS generation in DENV2 infected C6/36 mosquito cells in comparison with mock-infected cells. Additionally, a slight increase in MMP, and no differences in total ATP production or cell viability, was observed. These results suggest that increased ROS levels could be due to changes in MMP or mitochondrial function. Since MMP determines the energetic barrier for electron transport across the mitochondrial electron transport chain, it has been related to respiratory rates and the formation of mitochondrial ROS [5]. Importantly, these changes in mitochondrial function occur without differences in total ATP levels. Thus, we propose that the maintenance of total ATP levels has an important role in the maintenance of cell survival. However, it is important to measure the contribution of the different sources of ATP production.

Arboviruses establish persistent infection without apparent pathological effects in the mosquito host, ensuring life-long transmission to humans. These viruses are not eliminated from the vector as a result of the fine balance between virus replication and the antiviral host response [36], but the molecular mechanism remains to be characterized. During persistent infection, the cells are reprogrammed and become resistant to apoptosis. This resistance is often attributed to the modulation of MMP [37]. Therefore, our findings highlighting the changes in redox status of DENV infected cells might be of interest as it provides a wide platform for future studies to further explore and establish how persistent infection is achieved by arboviruses in vectors. Our finding is also in agreement with previous reports. For example, the protein X of Bornavirus colocalized to mitochondria, and inhibited apoptosis to promote persistence in infected cells [38]. Other pathways can help to explain the cell survival and viral persistence in mosquito cells. It is known that DENV infection produces ER stress with the activation of the unfolded protein response (UPR) pathway [39]. UPR is modulated by the induction of protein kinase RNA-like endoplasmic reticulum kinase (PERK), serine/threonine protein kinase/endoribonuclease (IRE1a) and the activating factor of transcription 6 (ATF6) [40]. Recently, it was reported that PERK is involved in determining survival in C6/36 mosquito cells by modulating protein translation [41]. 

During virus replication, cellular energy is required to generate new virions, and consequently, viruses modulate the energetic metabolism in different ways. X-protein of Hepatitis B virus (HBV) downregulates mitochondrial enzymes involved in ETC to increase the level of mitochondrial ROS and lipid peroxidation [42]. The Bovine Herpesvirus 1 replication is dependent on complex respiratory activity, specifically of complex II, IV, and the ATP synthase [43]. The respiratory syncytial virus induces the reduction of complex I activity, leading to decreased mitochondrial respiration and increased ROS [44,45]. In agreement with these findings, DENV infection induced changes in mitochondrial function. Spare respiratory capacity represents the ability of substrate supply and electron transport to respond to an increase in energetic demand [17]. We observed an increase in the spare capacity after 24 hpi (Figure 5F), in agreement with an increase in RCR at the same time point (Figure 6A). Increased RCR represents increased capacity for substrate oxidation and ATP turnover and a low proton leak. These findings demonstrate that mitochondria enhance their respiratory capacity and bioenergetic limit shortly after DENV infection, probably as a mechanism to withstand infection and avoid apoptosis. Maximal mitochondrial respiration was maintained at all times tested (Figure 5E), indicating that mitochondrial dysfunction was not found after DENV infection. No differences in mitochondrial proton leak were observed at any of the times tested, although a slight decrease could be observed at 24 h, probably related to increase RCR at this time, and returned to normal levels by 36 hpi. Finally, non-mitochondrial respiration increased after 36 hpi, probably indicating non-mitochondrial oxygen consumption by oxidases that could contribute to ROS production at this time point. Our data suggest an important role for mitochondrial and extra-mitochondrial ROS sources during the initial DENV infection since non-mitochondrial respiration was affected until 36 hpi and increased ROS production was found after 24 hpi. Mitochondria might contribute to early ROS production while an extra-mitochondrial ROS source, probably NADPH oxidase, could signal mitochondrial changes and a late mitochondrial contribution to ROS levels. It will be important to assess the individual sources contributing to ROS production after DENV infection in future studies.

Cellular energy is provided by important metabolic pathways including glycolysis, *β*-oxidation, the tricarboxylic cycle, and OXPHOS. Previous studies reported that DENV infection alters the expression of proteins involved in energy and lipid metabolism in human and mosquito hosts [46,47,48,49]. Fontaine et al. [50] reported the importance of glycolysis during DENV infection, in which they showed enhancement of glucose consumption and increased levels of the GLUT1 and HK2 protein during DENV infection in mammalian cells. The inhibition of the glycolytic pathway reduced DENV virion production. Interestingly, Japanese encephalitis virus infection increased VDAC localization in the outer membrane of the mitochondria and colocalized with GRP78 and VDAC in the ER [51]. Furthermore, a reduction in DENV infection has also been reported in cells lacking VDAC [52].

Mitochondrial dynamics respond to variations in cellular conditions and are promptly regulated to overcome physiological stress and maintain cellular homeostasis. In the past few years, mitochondrial function during viral infections has been studied, showing that viruses have developed different strategies to subvert and benefit from energy metabolism machinery and mitochondrial dynamics. In general, mitochondrial fitness is associated with the maintenance of a respiratory rate and increased fusion, while fission decreases respiratory function [27]. Cells lacking MFN1, MFN2 and OPA1 completely lacked mitochondrial fusion and showed severe cellular defects, including poor cell growth, loss of MMP, decreased cellular respiration, and apoptosis [53]. Thus, the expression of MFN1 and MFN2 is necessary to maintain cellular survival; MFN1/MFN2-deficient cells exhibited diminished ATP levels due to uncoupled mitochondria [54]. During infection, viruses like HCV, HVB, influenza A, measles virus, and others promote fission and mitophagy, whereas DENV, HIV, SARS-CoV, and Sendai virus have been shown to promote fusion in favor of viral replication and propagation by both inhibiting an innate immune response and maintaining cell viability [26].

Recently, it has been demonstrated that DENV infection induced mitochondrial elongation in a human cell line causing an imbalance in mitochondrial dynamics that promotes viral replication and diminished RIG-I-dependent interferon response [13,14]. Mitochondria from DENV-infected hepatic cells showed increased respiration and enhancement in ATP production; therefore, the induction of mitochondrial fission for overexpression of Drp1 reduced DENV replication [13]. Nevertheless, little is known about mitochondrial dynamics in insect cells. It was reported that mutation of OPA1 results in a decline in *Drosophila* lifespan by increasing ROS production and decreasing complex II and III activities [55]. Thus, depletion of MFN or OPA1 led to dysfunctional mitochondria, activation of target of rapamycin (TOR), and a marked accumulation of lipid droplets in *Drosophila* germ cells [56]. In flight muscle mitochondria of *Ae. aegypti* mosquitoes, blood-feeding triggers functional and structural changes, leading to the activation of both *MFN* and *OPA1*, resulting in mitochondrial fusion in tandem with a reduction in ROS production [57]. Our results revealed that mitochondria fusion could be involved in the maintenance of mitochondrial function in mosquito cells (C6/36) infected with DENV, since MFNs colocalized in the inner mitochondrial membrane with UCP2 and Drp1 were not found to be localized to the organelle. One important question raised by our data is whether mitochondrial fusion may directly impact dengue virus replication and/or affect innate immune response in mosquito cells.

In summary, we provide an overview of the effects of DENV2 infection on mitochondrial function in mosquito cells. Our results showed alterations in the redox metabolism and MMP without consequences in total cellular ATP levels or cellular viability. However, we observed decreased cellular and basal mitochondrial respiration upon DENV infection as well increased RCR at early time points after infection. Since RCR is related to a better mitochondrial function, our data indicates that DENV infection induces mitochondrial fitness, which probably has an effect on cellular survival and avoidance of apoptosis upon viral infection. These studies highlight the role of mitochondrial dynamics, redox metabolism, and energy metabolism in mosquito cells infected with DENV and opens up new avenues for future investigations.

## Figures and Tables

**Table 1:** Compounds used to analyze the ETC in mitochondria-C6/36 mock and DENV2-infected cells.

Compound	Concentration	Purpose
Digitonine	25 μg/mL	Cell permeabilization
Succinate	0.5 mM	Substrate Complex II
ADP	2.5 mM	Substrate Complex IV
Cytochrome c	2.2 mM	Mitochondrial electronic transporter between respiratory complexes III and IV
Oligomycin	5 μg/mL	State 4o respiratory activity
CCCP	0.2 mM	Maximal respiratory activity/State 3u
Sodium azide	2 mM	Inhibitor of ATP synthase

## Data Availability

Not applicable.
